# Is Early Enteral Nutrition Better for Postoperative Course in Esophageal Cancer Patients?

**DOI:** 10.3390/nu5093461

**Published:** 2013-09-03

**Authors:** Kazuaki Kobayashi, Yu Koyama, Shin-ichi Kosugi, Takashi Ishikawa, Kaoru Sakamoto, Hiroshi Ichikawa, Toshifumi Wakai

**Affiliations:** Division of Digestive & General Surgery, Niigata University Graduate School of Medical & Dental Sciences, 1-757 Asahimachi, Niigata 951-8510, Japan; E-Mails: kobachan0714@yahoo.co.jp (K.K.); sugishin@med.niigata-u.ac.jp (S.K.); takish@med.niigata-u.ac.jp (T.I.); kao_sakamoto@hotmail.com (K.S.); hichikawa-nii@med.niigata-u.ac.jp (H.I.); wakait@med.niigata-u.ac.jp (T.W.)

**Keywords:** early enteral nutrition, esophageal cancer, TPN, systematic inflammatory response syndrome

## Abstract

We retrospectively examined esophageal cancer patients who received enteral nutrition (EN) to clarify the validity of early EN compared with delayed EN. A total of 103 patients who underwent transthoracic esophagectomy with three-field lymphadenectomy for esophageal cancer were entered. Patients were divided into two groups; Group E received EN within postoperative day 3, and Group L received EN after postoperative day 3. The clinical factors such as days for first fecal passage, the dose of postoperative albumin infusion, differences of serum albumin value between pre- and postoperation, duration of systematic inflammatory response syndrome (SIRS), incidence of postoperative infectious complication, and use of total parenteral nutrition (TPN) were compared between the groups. The statistical analyses were performed using Mann-Whitney U test and Chi square test. The statistical significance was defined as *p* < 0.05. Group E showed fewer days for the first fecal passage (*p* < 0.01), lesser dose of postoperative albumin infusion (*p* < 0.01), less use of TPN (*p* < 0.01), and shorter duration of SIRS (*p* < 0.01). However, there was no significant difference in postoperative complications between the two groups. Early EN started within 3 days after esophagectomy. It is safe and valid for reduction of albumin infusion and TPN, for promoting early recovery of intestinal movement, and for early recovery from systemic inflammation.

## 1. Introduction

Several studies have elucidated the validity of early enteral nutrition (EN) in various patient groups including critically ill patients [1,2,3], head-injury patients [4,5], burns patients [6,7], trauma patients [8,9] and septic patients [10,11]. The efficacy of early enteral nutririon after surgery for patients who have undergone major surgery has been also proven [12,13,14,15,16,17]. These studies have proven the advantages of postoperative early EN as follows: lower incidence of septic complication [11], reduction of length of hospital stay, diminishing the degree of weight loss [15]. The term “early” was classically defined as EN started within 3 days after admission or surgery [18]; however, more recently, “early” has been defined as EN started within 48 or 24 h after admission or surgery [1].

On the other hand, transthoracic esophagectomy with 3-field lymphadenectomy (TTE-3FL) for esophageal cancer is one of the most radical and invasive surgeries among gastrointestinal surgical procedures. Past reports on early EN for surgery, described above [12,13,14,15,16,17], referred mainly to colorectal or gastrointestinal surgery. Therefore, the population of TTE-3FL was very small. In general, the patients who received TTE-3FL are not able to eat via mouth for a few days to one week after surgery. Therefore, postoperative enteral nutrition and/or parenteral nutrition after esophageal surgery is routine management. Recent reports have proven the advantages of early enteral nutrition, started within 24–48 h after esophagectomy for reduction of the length of hospital stay [19], reducing postoperative morbidity [20] and also reducing the rate of life-threatening complications [21]. However, because some studies have not shown any clinical benefits with routine postoperative EN after esophagectomy [22,23], the validity of early EN after esophagectomy has remained controversial [24].

The aim of the present retrospective study was to clarify the validity of early EN for postoperative course compared with delayed EN.

## 2. Patients and Methods

### 2.1. Patient Selection

The patients who underwent transthoracic esophagectomy with three-field lymphadenectomy for esophageal cancer at Niigata University Medical and Dental Hospital during 1996–2010 were entered into the present study. This study was a retrospective chart review, and a total of 103 patients were enrolled into the analysis. The data of patients were analyzed following approval from the Institutional Review Board for Clinical Research. There was no precise protocol for starting EN in our department; therefore, the date of EN initiation was decided individually according to the clinical experience of the doctors in charge of each patient, and also according to the condition of each patient. In the present study, we used the classical criteria of early EN as starting within 3 days after surgery in this study, according to the classical definition [18]. The patients were divided into two groups by the date they started EN; Group E contained the patients who received EN within postoperative day 3, and Group L contained the patients who received EN after postoperative day 3. EN was started with an initial dose of 200–250 mL of oligomeric or polymeric formula under 20–25 mL/h from jejunostomy. EN dose was gradually increased every 12–24 h if there were no problems related to EN, and reached a maximum dose 5–6 days after starting EN in both groups. Peripheral intravenous infusion of 4.3% glucose with electrolyte solutions were also performed to supply water and electrolyte in both groups. TPN was introduced if EN could not be started at postoperative day 5.

### 2.2. Clinical Assessment

Clinical factors such as: age, sex, tumor stage according to the tumor-node-metastasis classification of the International Union Against cancer (6th edition) [25], bowel movement recovery expressed as days for first fecal passage, the dose of postoperative albumin infusion used, difference of serum albumin value between day 7 and pre-operation (Δalb), duration of systematic inflammatory response syndrome (SIRS), incidence of postoperative complications, and use of total parenteral nutrition (TPN) were compared between group E and group L. SIRS was diagnosed according to the criteria of the ACCP-SCCM Consensus Conference Committee [26]. For the diagnosis, at least two of the following criteria had to be fulfilled: systolic blood pressure <90 mmHg, tachycardia >90/min, respiratory rate >20/min or peripheral arterial CO_2_ tension (PaCO_2_) <32 mmHg, temperature >38.0 °C or <36.0 °C, leukocytosis >12,000/μL or leukopenia <4000/μL or 10% immature (band) forms.

Postoperative complications were also retrospectively searched from patient records, and complications were divided into two categories: mechanical and infectious. Mechanical complication was defined as the complication directly due to failure of surgical procedure, and infectious complication was defined as the complication accompanying infection such as pneumonia, wound infection, enteritis and/or sepsis in this study. Recurrent nerve palsy indicated as vocal cord function was assessed by laryngofiberscopy in all patients, regardless of the presence or absence of hoarseness, as previously described in [27].

### 2.3. Statistical Analysis

The statistical analyses were performed using the Mann-Whitney *U* test and adjusted Chi-square test. The Exact *Chi-square* test was also used if individual cell size was less than 5 counts. The statistical significance was defined as *p* < 0.05.

## 3. Results

### 3.1. Pre- and Perioperative Clinical Features

Among a total of 103 patients, 42 patients categorized in Group E, and 61 patients categorized in Group L (Table 1). Both Group E and Group L were comparable in mean age, sex, preoperative nutritional conditions expressed by body mass index (BMI), body weight and serum albumin value, However, our results in this study were analyzed under a mixed, male and female population; therefore, gender might have some effects on our results. The distribution of the clinical stage of cancer was not similar between Group E and Group L (Group E *versus* Group L, stage 0: 2 *vs*. 0; stage I: 15 *vs*. 10; stage IIA: 7 *vs*. 5; stage IIB: 2 *vs*. 11; stage III: 8 *vs*. 19; stage IVA: 3 *vs*. 4; stage IVB: 5 *vs*. 12). However, if an earlier stage (stage 0–IIB) is compared with a more advanced stage (stage III–IVB), there was no significant difference in distribution of the stages between Group E and Group L, as shown in Table 1. The status of preoperative chemotherapy was significantly different: Group E patients received preoperative chemotherapy more frequently than Group L patients (*p* < 0.01). Postoperative TPN was more frequently used in Group L compared with Group E (*p* < 0.01).

**Table 1:** Pre- and Perioperative Clinical Features: Early *versus* Late Enteral Nutrition (EN) (*n* = 103).

		Group E (*n* = 43)	Group L (*n* = 61)	*p* value (Mann-Whitney test)
Age (years) ^a^		61.5 ± 6.6	62.7 ± 6.7	NS
BMI (kg/m^2^) ^a^		21.8 ± 3.1	21.2 ± 2.6	NS
Body weight (kg) ^a^		57.7 ± 9.8	55.3 ± 9.4	NS
Albumin (mg/dL) ^a^		4.0 ± 0.4	4.0 ± 0.4	NS
Surgery time (min) ^a^		470.1 ± 83.2	481.1 ± 80.1	NS
				*p* value (Chi square test)
Sex				NS
	Male	36	53	
	Female	6	8	
Stage				NS
	0–IIB	26	26	
	III–IVB	16	35	
Preoperative chemotherapy				<0.01
	No	22	50	
	Yes	20	11	
Postopertive TPN use				<0.01
	No	32	10	
	Yes	10	51	

^a^ Data are mean ± standard deviation; TPN: total parenteral nutrition.

### 3.2. Postoperative Complications and Mortality

Postoperative complications were observed in 87 patients (84.4%) and there was no significant difference in whole complications between Group E and Group L. Furthermore, complications were categorized into two groups: mechanical and infectious. These two groups were comparable in whole mechanical complication. However, there was significant difference in the frequency of anastomotic dehiscence: anastomotic dehiscence was observed more frequently in Group E compared with Group L (*p* < 0.01). The frequency of recurrent nerve palsy, observed in our series, was quite high in both groups; however, almost recurrent nerve palsy was subclinical and transient. On the other hand, there was no significant difference in infectious complication, including pneumonia, wound infection, enteritis and sepsis. Perioperative death was observed in only one patient in Group E: aortic rupture occurred at postopretative day 8. The mortality of the two groups was comparable (Table 2).

**Table 2:** Postoperative complication and Mortality: Early *versus* Late EN (*n* = 103).

	Group E (*n* = 42)	Group L (*n* = 61)	*p* value
Postoperative complications	36	51	NS
Mechanical	22	30	NS
Recurrent nerve palsy	22	41	NS
Anastomotic dehiscence	14	6	<0.01
Tracheal damage	1	2	NS
Aortic rupture	1	0	NS
Infectious	15	21	NS
Pneumonia	7	10	NS
Wound infection	6	9	NS
Enteritis/intestinal ischemia	0	5	NS
Sepsis	3	3	NS
Mortality	1	0	NS

### 3.3. Postoperative Outcomes Comparing Early EN with Late EN

Postoperative EN calorie (kcal/kg) supplied at maximum was significantly higher in group E compared with group L (28.5 *versus* 16.1; *p* < 0.01). Bowel movement recovery was observed significantly earlier in Group E (*p* < 0.01) and also the duration of SIRS and respirator management were significantly shorter in Group E (*p* < 0.01). The volume of albumin infusion was significantly smaller in Group E (*p* < 0.01). However, the decreased value of serum albumin (Δalb) was more prominent in Group E compared with Group L (*p* < 0.01) (Table 3). The length of hospital stay was, however, slightly shorter in Group E compared with Group L (*p* < 0.05).

**Table 3:** Postoperative outcomes: Early *versus* Late EN (*n* = 103).

	Group E (*n* = 42)	Group L (*n* = 61)	*p* value
EN Calorie (kcal/kg) ^a^	28.5 ± 8.5	16.1 ± 5.4	<0.01
Bowel movement recovery (day) ^a^	5.2 ± 1.6	7.4 ± 2.3	<0.01
Albumin infusion (mL) ^a^	83 ± 62	169 ± 101	<0.01
∆albumin (mg/dL) ^a^	−1.2 ± 0.5	−0.8 ± 0.5	<0.01
SIRS duration (day) ^a^	4.0 ± 5.1	6.2 ± 5.8	<0.01
Respirator duration (day) ^a^	3.7 ± 7.3	7.0 ± 5.1	<0.01
LOH (day) ^a^	54.2 ± 52.0	66.3 ± 54.1	<0.05

^a^ Data are mean ± standard deviation; ∆albumin: difference of serum albumin value between day 7 and pre-operation; SIRS: systematic inflammatory response syndrome; LOH: length of hospital stay.

## 4. Discussion

Esophageal cancer patients are frequently malnourished due to esophageal stenosis, because of: their habits, preoperative systemic chemotherapy or the systemic effect of their neoplasm [28]. However, preoperative nutritional status, expressed by BMI, body weight and serum albumin value, showed quite good nutritional preference, and the preoperative nutritional condition of each group was comparable in this study. Surgical procedure also seemed to be comparable between the two groups because there was no significant difference in operation time between the two groups. Therefore, we think the level of surgical stress will be comparable between Group E and Group L in the present study.

In the present study, there was historical bias in the start date of enteral feeding and the rate of preoperative chemotherapy; the average start date of EN was later in the patients during 1996–2000 (average start day of EN during 1996–2000 *versus* 2001–2010: 7.1 days *versus* 3.3 days, *p* < 0.01), in contrast with the rate of preoperative chemotherapy, which was higher in the patients during 2001–2010.

Although there were some reports that early EN, started within 24 h after esophagectomy, has shown an advantage in reducing postoperative morbidity or life-threatening complications [20,21], we could not find an advantage in reducing complications by using “early” EN in the present study. The discrepancy of the result on morbidity between past studies and ours might be due to the difference in definition of “early” EN. We used the criteria at within 3 days, and this might be why we could not find an advantage in reducing morbidity in Group E. However, “early” EN was safely and effectively performed in our series, because the patients of Group E showed significantly earlier recovery from SIRS, and also showed shorter respirator use compared with the patients of Group L, while there seemed to be a similar level of surgical stress. Moreover, our study revealed the advantage of early EN in the recovery of intestinal movement, indicated by significantly earlier first fecal passage in Group E. These results revealed that “early” EN, even if started within 3 days after surgery, was enough to take advantage of earlier recovery from systemic inflammation and respiratory disorder.

Our study has shown that the frequency of recurrent nerve palsy was quite high in both groups; however, recurrent nerve palsy was strictly assessed by endoscopically in our department, and this seemed to be the reason for the high frequency of recurrent nerve palsy. Indeed, cases of recurrent nerve palsy in this study were not prominent.

The frequency of anastomotic dehiscence was significantly higher in Group E in this study. A possible explanation for this is that the patients in Group E received damage by preoperative chemotherapy, which was more frequently observed in Group E compared with Group L. The damage to cell recycling, vascularization, and tissue regeneration may affect the anastomotic failure, which was more frequent in Group E.

Several factors may potentially impact on the frequency of postoperative complication in our study because some factors of patient background, such as preoperative stage or preoperative therapy, were quite different. Therefore, it may be desirable to perform mulitvariate analysis to search major factors which have an impact on postoperative complication. However, the aim of this study was to clarify the validity of early EN for postoperative course compared with delayed EN; it seemed to be unnecessary to search for the factors which have an impact on postoperative complication other than EN.

The decrease of serum albumin at postoperative day 7 was significantly prominent in Group E. On the other hand, albumin infusion was significantly less in Group E. Moreover, there was no significantly difference in morbidity and mortality between group E and group L, as described above. These results showed that early EN could have the advantage of reducing albumin infusion, which means less expensive treatment. In addition, postoperative TPN was less frequently used in Group E compared with Group L.

Finally, most patients in this study were male. Females constituted a small, but, however, not negligible part of the study. Gender may affect our results in terms of: clinical factors, postoperative complications and outcomes of patients. Therefore, we have re-analyzed by using the data from only the male population. The results from 89 males in this study still showed significantly fewer days for the first fecal passage, a lesser dose of postoperative albumin infusion, less use of TPN, shorter duration of SIRS, shorter duration under respirator, and shorter length of hospital stay in Group E compared with Group L (data not shown). Again, gender may affect our results, however, we think that our results in this study will be reliable enough, if analyzed under a mixed male and female population.

## 5. Conclusions

Early EN started within 3 days is safe and valid for postoperative esophageal cancer patients and has advantages in reducing the use of albumin infusion and TPN, for promoting early recovery of intestinal movement, and for early recovery from systemic inflammation.
